# CD40 expression in renal cell carcinoma correlates with tumor apoptosis, CD8 T cell frequency and patient survival

**DOI:** 10.1186/2051-1426-1-S1-P65

**Published:** 2013-11-07

**Authors:** Jonathan M Weiss, Gregory Alvord, Octavio A Quinones, Jimmy K Stauffer, Robert H Wiltrout

**Affiliations:** 1 Cancer and Inflammation Program, NCI Frederick, CCR, Frederick, MD, USA; 2 Statistical Consulting, DMS, Frederick National Laboratory for Cancer Research, Frederick, MD, USA

## Purpose

To investigate the relationship between tumor-associated CD40 expression, RCC patient survival and tumor stage.

## Experimental design

The expression of CD40, TUNEL and CD8 in human renal cell carcinomas was analyzed by immunohistochemistry. Computer-assisted quantitation of protein expression was used to correlate results with patient survival and tumor stage.

## Results

We show for the first time that tumor-associated CD40 expression correlates with prolonged survival in RCC patients. CD40 also correlated with TUNEL and CD8 staining in tumor tissues. No correlation was observed between CD40 and tumor stage.

## Conclusions

Our results suggest CD40 may be a prognostic biomarker indicative of prolonged RCC patient survival. Strategies that up-regulate CD40 expression in some RCC patients might lead to improved survival. The further development and use of agonistic CD40 antibodies for treatment of RCC is warranted.

